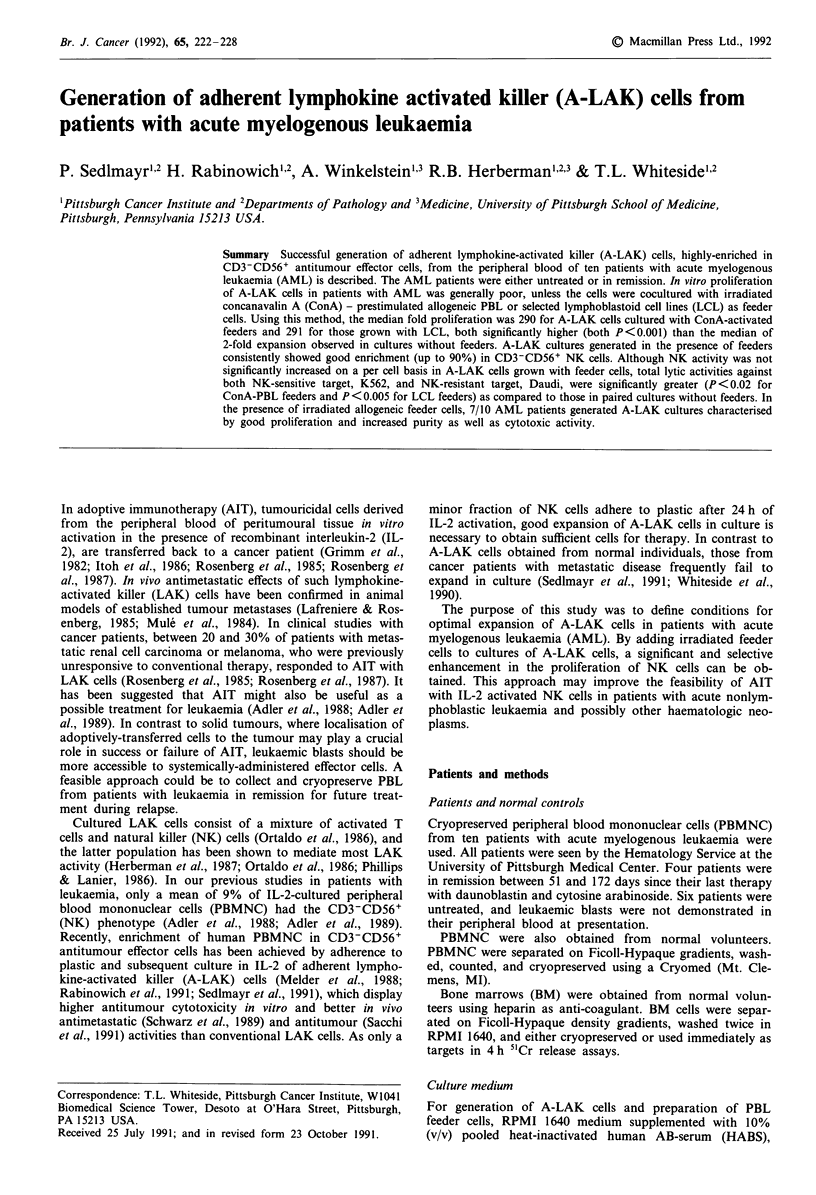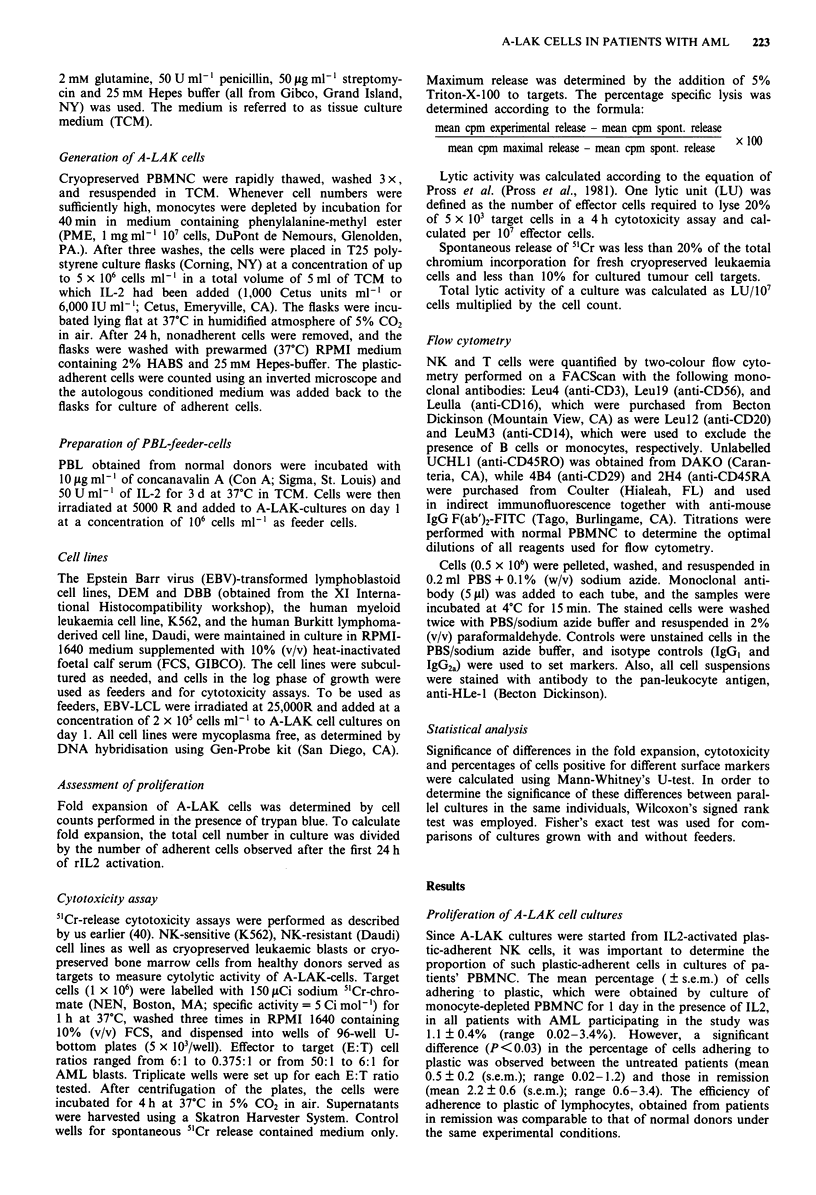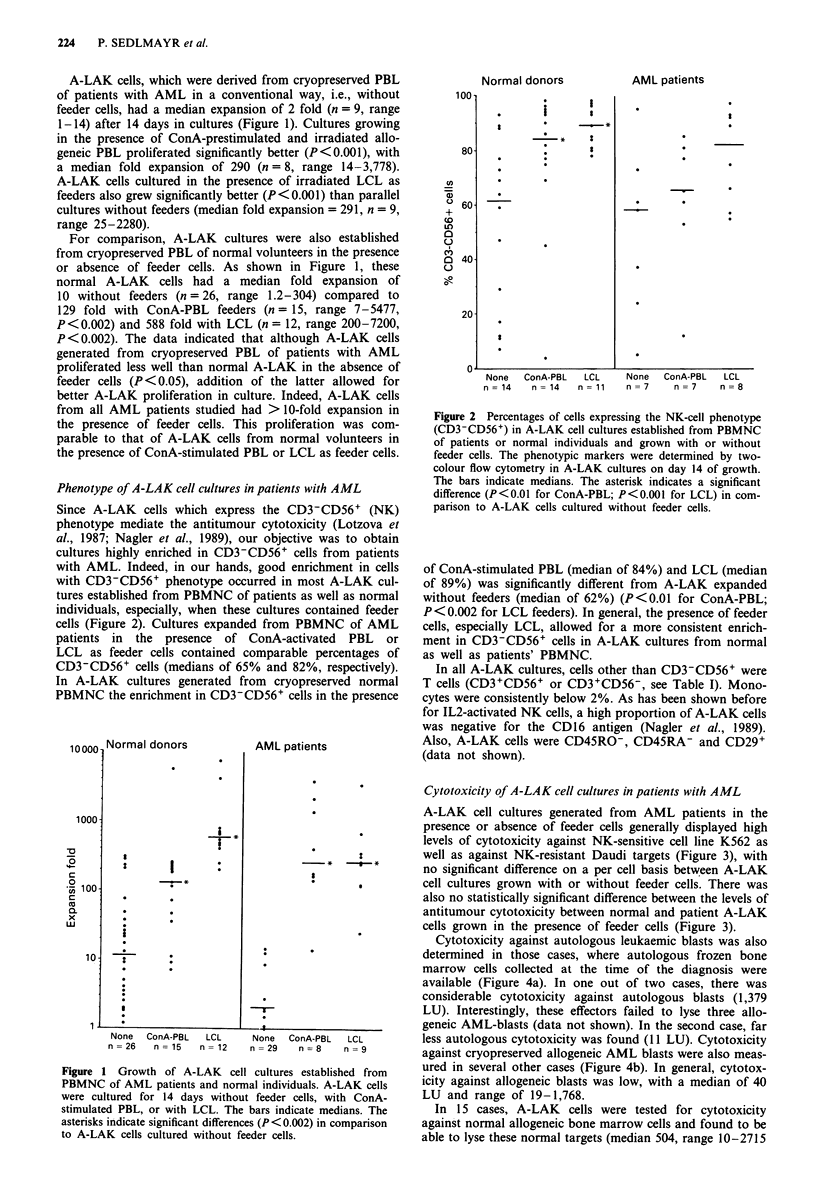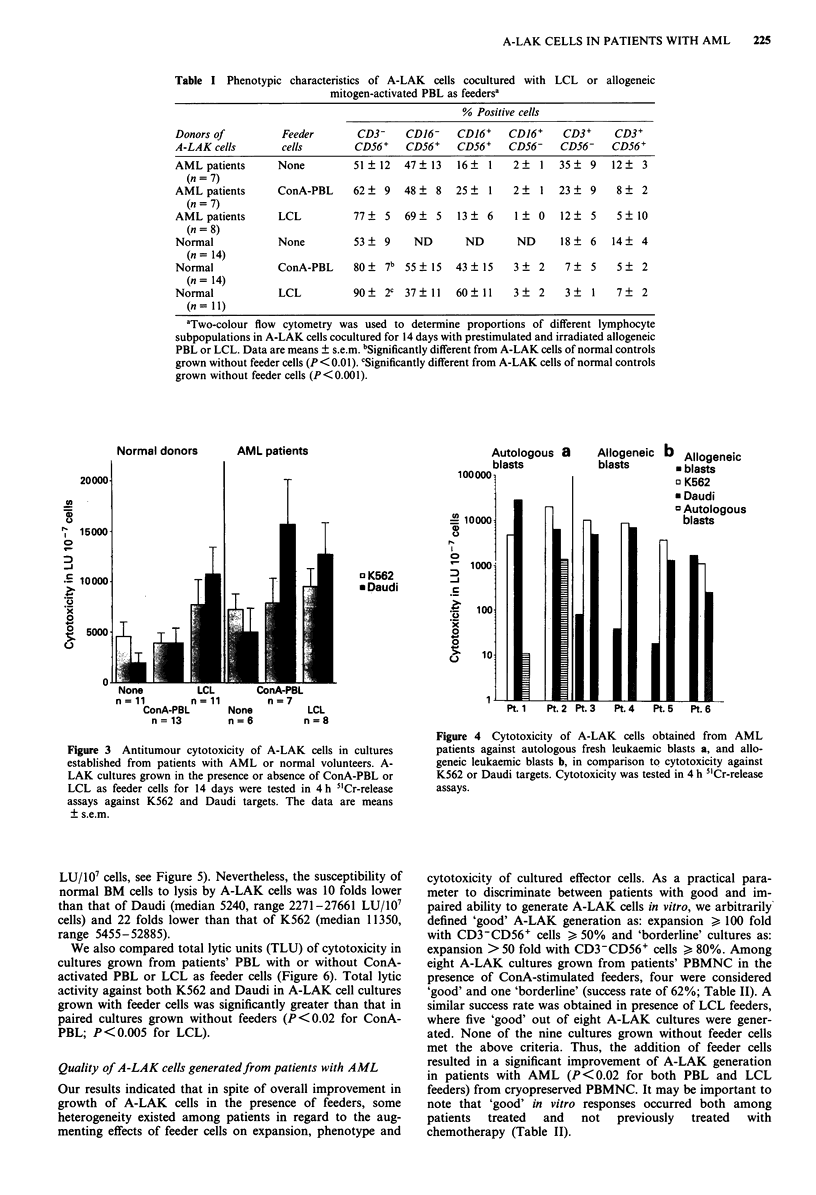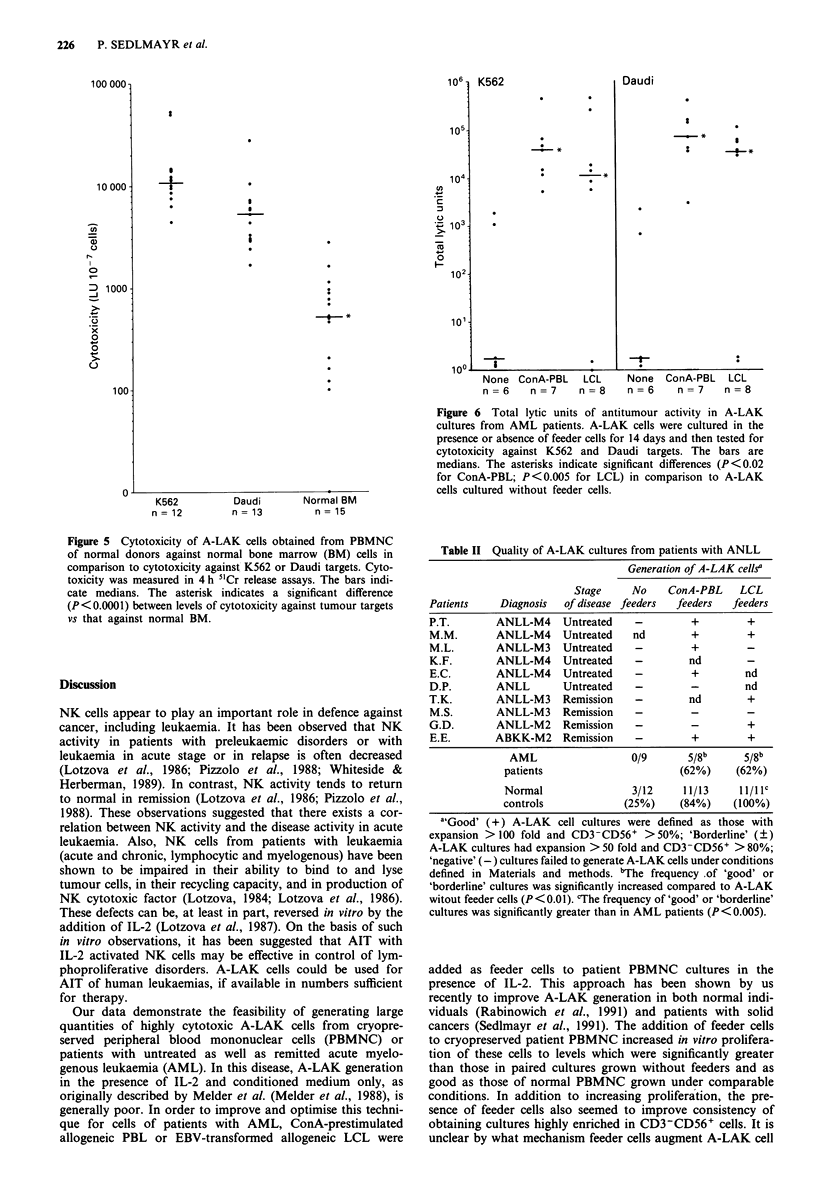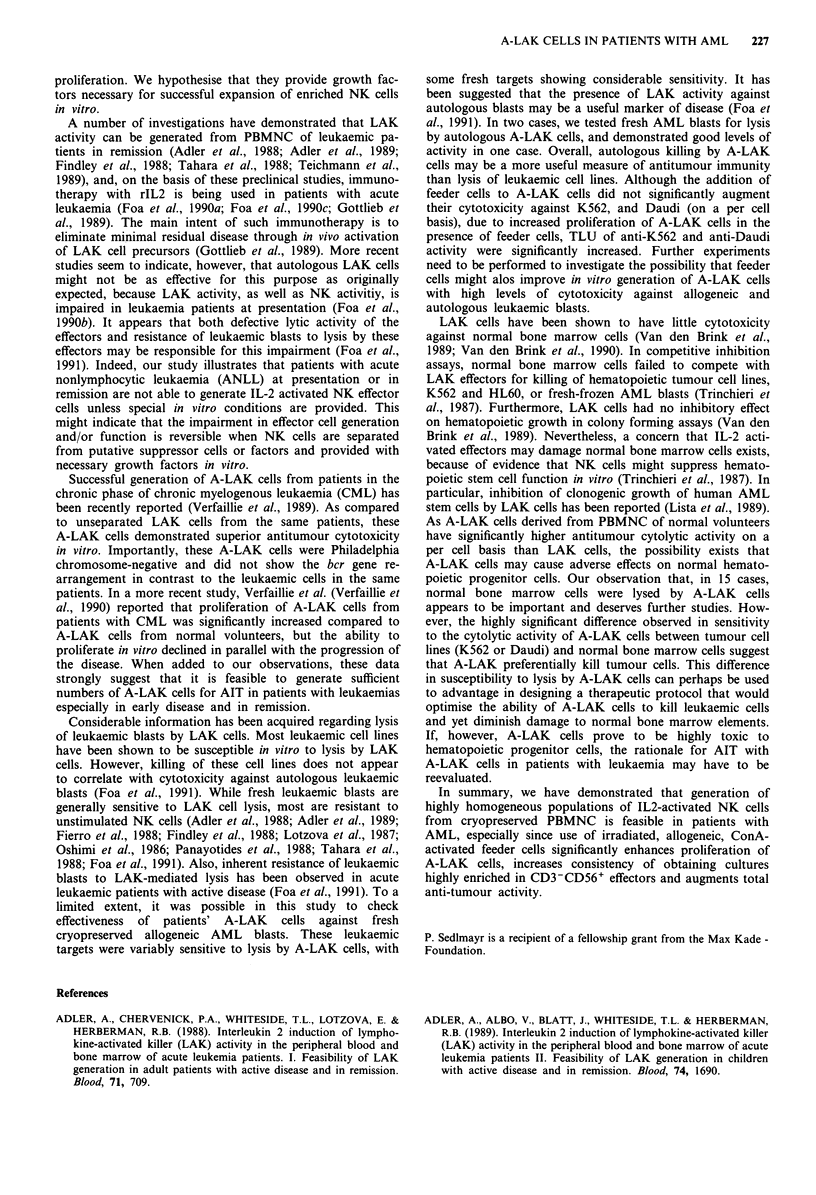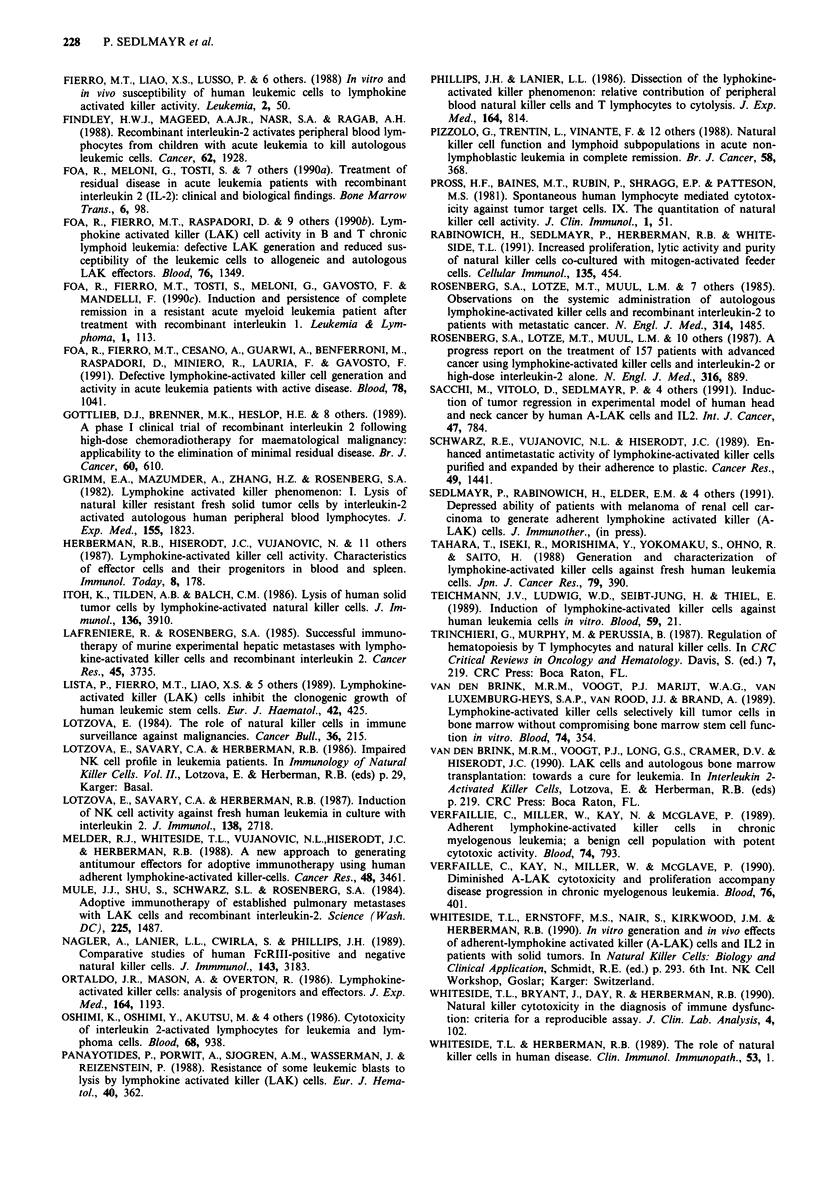# Generation of adherent lymphokine activated killer (A-LAK) cells from patients with acute myelogenous leukaemia.

**DOI:** 10.1038/bjc.1992.45

**Published:** 1992-02

**Authors:** P. Sedlmayr, H. Rabinowich, A. Winkelstein, R. B. Herberman, T. L. Whiteside

**Affiliations:** Pittsburgh Cancer Institute, University of Pittsburgh School of Medicine, Pennsylvania.

## Abstract

Successful generation of adherent lymphokine-activated killer (A-LAK) cells, highly-enriched in CD3-CD56+ antitumour effector cells, from the peripheral blood of ten patients with acute myelogenous leukaemia (AML) is described. The AML patients were either untreated or in remission. In vitro proliferation of A-LAK cells in patients with AML was generally poor, unless the cells were cocultured with irradiated concanavalin A (ConA)--prestimulated allogeneic PBL or selected lymphoblastoid cell lines (LCL) as feeder cells. Using this method, the median fold proliferation was 290 for A-LAK cells cultured with ConA-activated feeders and 291 for those grown with LCL, both significantly higher (both P less than 0.001) than the median of 2-fold expansion observed in cultures without feeders. A-LAK cultures generated in the presence of feeders consistently showed good enrichment (up to 90%) in CD3-CD56+ NK cells. Although NK activity was not significantly increased on a per cell basis in A-LAK cells grown with feeder cells, total lytic activities against both NK-sensitive target, K562, and NK-resistant target, Daudi, were significantly greater (P less than 0.02 for ConA-PBL feeders and P less than 0.005 for LCL feeders) as compared to those in paired cultures without feeders. In the presence of irradiated allogeneic feeder cells, 7/10 AML patients generated A-LAK cultures characterised by good proliferation and increased purity as well as cytotoxic activity.


Br. J. Cancer (1992), 65, 222-228 © Macmillan Press Ltd., 1992

### Generation of adherent lymphokine activated killer (A-LAK) cells from patients with acute myelogenous leukaemia

P. Sedlmayr[1,2] H. Rabinowich[1,2], A. Winkelstein[1,3] R.B. Herberman[1,2,3] & T.L. Whiteside[1,2]

[1]Pittsburgh Cancer Institute and [2]Departments of Pathology and [3]Medicine, University of Pittsburgh School of Medicine, Pittsburgh, Pennsylvania 15213 USA.

**Summary** Successful generation of adherent lymphokine-activated killer (A-LAK) cells, highly-enriched in $CD3^-CD56^+$ antitumour effector cells, from the peripheral blood of ten patients with acute myelogenous leukaemia (AML) is described. The AML patients were either untreated or in remission. *In vitro* proliferation of A-LAK cells in patients with AML was generally poor, unless the cells were cocultured with irradiated concanavalin A (ConA) – prestimulated allogeneic PBL or selected lymphoblastoid cell lines (LCL) as feeder cells. Using this method, the median fold proliferation was 290 for A-LAK cells cultured with ConA-activated feeders and 291 for those grown with LCL, both significantly higher (both $P<0.001$) than the median of 2-fold expansion observed in cultures without feeders. A-LAK cultures generated in the presence of feeders consistently showed good enrichment (up to 90%) in $CD3^-CD56^+$ NK cells. Although NK activity was not significantly increased on a per cell basis in A-LAK cells grown with feeder cells, total lytic activities against both NK-sensitive target, K562, and NK-resistant target, Daudi, were significantly greater ($P<0.02$ for ConA-PBL feeders and $P<0.005$ for LCL feeders) as compared to those in paired cultures without feeders. In the presence of irradiated allogeneic feeder cells, 7/10 AML patients generated A-LAK cultures characterised by good proliferation and increased purity as well as cytotoxic activity.

In adoptive immunotherapy (AIT), tumouricidal cells derived from the peripheral blood of peritumoural tissue *in vitro* activation in the presence of recombinant interleukin-2 (IL-2), are transferred back to a cancer patient (Grimm *et al.*, 1982; Itoh *et al.*, 1986; Rosenberg *et al.*, 1985; Rosenberg *et al.*, 1987). *In vivo* antimetastatic effects of such lymphokine-activated killer (LAK) cells have been confirmed in animal models of established tumour metastases (Lafreniere & Rosenberg, 1985; Mulé *et al.*, 1984). In clinical studies with cancer patients, between 20 and 30% of patients with metastatic renal cell carcinoma or melanoma, who were previously unresponsive to conventional therapy, responded to AIT with LAK cells (Rosenberg *et al.*, 1985; Rosenberg *et al.*, 1987). It has been suggested that AIT might also be useful as a possible treatment for leukaemia (Adler *et al.*, 1988; Adler *et al.*, 1989). In contrast to solid tumours, where localisation of adoptively-transferred cells to the tumour may play a crucial role in success or failure of AIT, leukaemic blasts should be more accessible to systemically-administered effector cells. A feasible approach could be to collect and cryopreserve PBL from patients with leukaemia in remission for future treatment during relapse.

Cultured LAK cells consist of a mixture of activated T cells and natural killer (NK) cells (Ortaldo *et al.*, 1986), and the latter population has been shown to mediate most LAK activity (Herberman *et al.*, 1987; Ortaldo *et al.*, 1986; Phillips & Lanier, 1986). In our previous studies in patients with leukaemia, only a mean of 9% of IL-2-cultured peripheral blood mononuclear cells (PBMNC) had the $CD3^-CD56^+$ (NK) phenotype (Adler *et al.*, 1988; Adler *et al.*, 1989). Recently, enrichment of human PBMNC in $CD3^-CD56^+$ antitumour effector cells has been achieved by adherence to plastic and subsequent culture in IL-2 of adherent lymphokine-activated killer (A-LAK) cells (Melder *et al.*, 1988; Rabinowich *et al.*, 1991; Sedlmayr *et al.*, 1991), which display higher antitumour cytotoxicity *in vitro* and better *in vivo* antimetastatic (Schwarz *et al.*, 1989) and antitumour (Sacchi *et al.*, 1991) activities than conventional LAK cells. As only a minor fraction of NK cells adhere to plastic after 24 h of IL-2 activation, good expansion of A-LAK cells in culture is necessary to obtain sufficient cells for therapy. In contrast to A-LAK cells obtained from normal individuals, those from cancer patients with metastatic disease frequently fail to expand in culture (Sedlmayr *et al.*, 1991; Whiteside *et al.*, 1990).

The purpose of this study was to define conditions for optimal expansion of A-LAK cells in patients with acute myelogenous leukaemia (AML). By adding irradiated feeder cells to cultures of A-LAK cells, a significant and selective enhancement in the proliferation of NK cells can be obtained. This approach may improve the feasibility of AIT with IL-2 activated NK cells in patients with acute nonlymphoblastic leukaemia and possibly other haematologic neoplasms.

### Patients and methods

### Patients and normal controls

Cryopreserved peripheral blood mononuclear cells (PBMNC) from ten patients with acute myelogenous leukaemia were used. All patients were seen by the Hematology Service at the University of Pittsburgh Medical Center. Four patients were in remission between 51 and 172 days since their last therapy with daunoblastin and cytosine arabinoside. Six patients were untreated, and leukaemic blasts were not demonstrated in their peripheral blood at presentation.

PBMNC were also obtained from normal volunteers. PBMNC were separated on Ficoll-Hypaque gradients, washed, counted, and cryopreserved using a Cryomed (Mt. Clemens, MI).

Bone marrows (BM) were obtained from normal volunteers using heparin as anti-coagulant. BM cells were separated on Ficoll-Hypaque density gradients, washed twice in RPMI 1640, and either cryopreserved or used immediately as targets in 4 h $^{51}Cr$ release assays.

### Culture medium

For generation of A-LAK cells and preparation of PBL feeder cells, RPMI 1640 medium supplemented with 10% (v/v) pooled heat-inactivated human AB-serum (HABS),

Correspondence: T.L. Whiteside, Pittsburgh Cancer Institute, W1041 Biomedical Science Tower, Desoto at O'Hara Street, Pittsburgh, PA 15213 USA.
Received 25 July 1991; and in revised form 23 October 1991.

2 mM glutamine, 50 U $ml^{-1}$ penicillin, 50 $\mu$g $ml^{-1}$ streptomycin and 25 mM Hepes buffer (all from Gibco, Grand Island, NY) was used. The medium is referred to as tissue culture medium (TCM).

*Generation of A-LAK cells*

Cryopreserved PBMNC were rapidly thawed, washed 3×, and resuspended in TCM. Whenever cell numbers were sufficiently high, monocytes were depleted by incubation for 40 min in medium containing phenylalanine-methyl ester (PME, 1 mg $ml^{-1}$ $10^7$ cells, DuPont de Nemours, Glenolden, PA.). After three washes, the cells were placed in T25 polystyrene culture flasks (Corning, NY) at a concentration of up to $5 \times 10^6$ cells $ml^{-1}$ in a total volume of 5 ml of TCM to which IL-2 had been added (1,000 Cetus units $ml^{-1}$ or 6,000 IU $ml^{-1}$; Cetus, Emeryville, CA). The flasks were incubated lying flat at 37°C in humidified atmosphere of 5% $CO_2$ in air. After 24 h, nonadherent cells were removed, and the flasks were washed with prewarmed (37°C) RPMI medium containing 2% HABS and 25 mM Hepes-buffer. The plastic-adherent cells were counted using an inverted microscope and the autologous conditioned medium was added back to the flasks for culture of adherent cells.

*Preparation of PBL-feeder-cells*

PBL obtained from normal donors were incubated with 10 $\mu$g $ml^{-1}$ of concanavalin A (Con A; Sigma, St. Louis) and 50 U $ml^{-1}$ of IL-2 for 3 d at 37°C in TCM. Cells were then irradiated at 5000 R and added to A-LAK-cultures on day 1 at a concentration of $10^6$ cells $ml^{-1}$ as feeder cells.

*Cell lines*

The Epstein Barr virus (EBV)-transformed lymphoblastoid cell lines, DEM and DBB (obtained from the XI International Histocompatibility workshop), the human myeloid leukaemia cell line, K562, and the human Burkitt lymphoma-derived cell line, Daudi, were maintained in culture in RPMI-1640 medium supplemented with 10% (v/v) heat-inactivated foetal calf serum (FCS, GIBCO). The cell lines were subcultured as needed, and cells in the log phase of growth were used as feeders and for cytotoxicity assays. To be used as feeders, EBV-LCL were irradiated at 25,000R and added at a concentration of $2 \times 10^5$ cells $ml^{-1}$ to A-LAK cell cultures on day 1. All cell lines were mycoplasma free, as determined by DNA hybridisation using Gen-Probe kit (San Diego, CA).

*Assessment of proliferation*

Fold expansion of A-LAK cells was determined by cell counts performed in the presence of trypan blue. To calculate fold expansion, the total cell number in culture was divided by the number of adherent cells observed after the first 24 h of rIL2 activation.

*Cytotoxicity assay*

$^{51}$Cr-release cytotoxicity assays were performed as described by us earlier (40). NK-sensitive (K562), NK-resistant (Daudi) cell lines as well as cryopreserved leukaemic blasts or cryopreserved bone marrow cells from healthy donors served as targets to measure cytolytic activity of A-LAK-cells. Target cells ($1 \times 10^6$) were labelled with 150 $\mu$Ci sodium $^{51}$Cr-chromate (NEN, Boston, MA; specific activity = 5 Ci $mol^{-1}$) for 1 h at 37°C, washed three times in RPMI 1640 containing 10% (v/v) FCS, and dispensed into wells of 96-well U-bottom plates ($5 \times 10^3$/well). Effector to target (E:T) cell ratios ranged from 6:1 to 0.375:1 or from 50:1 to 6:1 for AML blasts. Triplicate wells were set up for each E:T ratio tested. After centrifugation of the plates, the cells were incubated for 4 h at 37°C in 5% $CO_2$ in air. Supernatants were harvested using a Skatron Harvester System. Control wells for spontaneous $^{51}$Cr release contained medium only. Maximum release was determined by the addition of 5% Triton-X-100 to targets. The percentage specific lysis was determined according to the formula:

$$\frac{\text{mean cpm experimental release} - \text{mean cpm spont. release}}{\text{mean cpm maximal release} - \text{mean cpm spont. release}} \times 100$$

Lytic activity was calculated according to the equation of Pross *et al.* (Pross *et al.*, 1981). One lytic unit (LU) was defined as the number of effector cells required to lyse 20% of $5 \times 10^3$ target cells in a 4 h cytotoxicity assay and calculated per $10^7$ effector cells.

Spontaneous release of $^{51}$Cr was less than 20% of the total chromium incorporation for fresh cryopreserved leukaemia cells and less than 10% for cultured tumour cell targets.

Total lytic activity of a culture was calculated as LU/$10^7$ cells multiplied by the cell count.

*Flow cytometry*

NK and T cells were quantified by two-colour flow cytometry performed on a FACScan with the following monoclonal antibodies: Leu4 (anti-CD3), Leu19 (anti-CD56), and Leulla (anti-CD16), which were purchased from Becton Dickinson (Mountain View, CA) as were Leu12 (anti-CD20) and LeuM3 (anti-CD14), which were used to exclude the presence of B cells or monocytes, respectively. Unlabelled UCHL1 (anti-CD45RO) was obtained from DAKO (Caranteria, CA), while 4B4 (anti-CD29) and 2H4 (anti-CD45RA were purchased from Coulter (Hialeah, FL) and used in indirect immunofluorescence together with anti-mouse IgG F(ab')$_2$-FITC (Tago, Burlingame, CA). Titrations were performed with normal PBMNC to determine the optimal dilutions of all reagents used for flow cytometry.

Cells ($0.5 \times 10^6$) were pelleted, washed, and resuspended in 0.2 ml PBS + 0.1% (w/v) sodium azide. Monoclonal antibody (5 $\mu$l) was added to each tube, and the samples were incubated at 4°C for 15 min. The stained cells were washed twice with PBS/sodium azide buffer and resuspended in 2% (v/v) paraformaldehyde. Controls were unstained cells in the PBS/sodium azide buffer, and isotype controls ($IgG_1$ and $IgG_{2a}$) were used to set markers. Also, all cell suspensions were stained with antibody to the pan-leukocyte antigen, anti-HLe-1 (Becton Dickinson).

*Statistical analysis*

Significance of differences in the fold expansion, cytotoxicity and percentages of cells positive for different surface markers were calculated using Mann-Whitney's U-test. In order to determine the significance of these differences between parallel cultures in the same individuals, Wilcoxon's signed rank test was employed. Fisher's exact test was used for comparisons of cultures grown with and without feeders.

### Results

*Proliferation of A-LAK cell cultures*

Since A-LAK cultures were started from IL2-activated plastic-adherent NK cells, it was important to determine the proportion of such plastic-adherent cells in cultures of patients' PBMNC. The mean percentage (± s.e.m.) of cells adhering to plastic, which were obtained by culture of monocyte-depleted PBMNC for 1 day in the presence of IL2, in all patients with AML participating in the study was 1.1 ± 0.4% (range 0.02–3.4%). However, a significant difference ($P < 0.03$) in the percentage of cells adhering to plastic was observed between the untreated patients (mean 0.5 ± 0.2 (s.e.m.); range 0.02–1.2) and those in remission (mean 2.2 ± 0.6 (s.e.m.); range 0.6–3.4). The efficiency of adherence to plastic of lymphocytes, obtained from patients in remission was comparable to that of normal donors under the same experimental conditions.

A-LAK cells, which were derived from cryopreserved PBL of patients with AML in a conventional way, i.e., without feeder cells, had a median expansion of 2 fold ($n = 9$, range 1–14) after 14 days in cultures (Figure 1). Cultures growing in the presence of ConA-prestimulated and irradiated allogeneic PBL proliferated significantly better ($P < 0.001$), with a median fold expansion of 290 ($n = 8$, range 14–3,778). A-LAK cells cultured in the presence of irradiated LCL as feeders also grew significantly better ($P < 0.001$) than parallel cultures without feeders (median fold expansion = 291, $n = 9$, range 25–2280).

For comparison, A-LAK cultures were also established from cryopreserved PBL of normal volunteers in the presence or absence of feeder cells. As shown in Figure 1, these normal A-LAK cells had a median fold expansion of 10 without feeders ($n = 26$, range 1.2–304) compared to 129 fold with ConA-PBL feeders ($n = 15$, range 7–5477, $P < 0.002$) and 588 fold with LCL ($n = 12$, range 200–7200, $P < 0.002$). The data indicated that although A-LAK cells generated from cryopreserved PBL of patients with AML proliferated less well than normal A-LAK in the absence of feeder cells ($P < 0.05$), addition of the latter allowed for better A-LAK proliferation in culture. Indeed, A-LAK cells from all AML patients studied had >10-fold expansion in the presence of feeder cells. This proliferation was comparable to that of A-LAK cells from normal volunteers in the presence of ConA-stimulated PBL or LCL as feeder cells.

*Phenotype of A-LAK cell cultures in patients with AML*

Since A-LAK cells which express the $CD3^-CD56^+$ (NK) phenotype mediate the antitumour cytotoxicity (Lotzova *et al.*, 1987; Nagler *et al.*, 1989), our objective was to obtain cultures highly enriched in $CD3^-CD56^+$ cells from patients with AML. Indeed, in our hands, good enrichment in cells with $CD3^-CD56^+$ phenotype occurred in most A-LAK cultures established from PBMNC of patients as well as normal individuals, especially, when these cultures contained feeder cells (Figure 2). Cultures expanded from PBMNC of AML patients in the presence of ConA-activated PBL or LCL as feeder cells contained comparable percentages of $CD3^-CD56^+$ cells (medians of 65% and 82%, respectively). In A-LAK cultures generated from cryopreserved normal PBMNC the enrichment in $CD3^-CD56^+$ cells in the presence of ConA-stimulated PBL (median of 84%) and LCL (median of 89%) was significantly different from A-LAK expanded without feeders (median of 62%) ($P < 0.01$ for ConA-PBL; $P < 0.002$ for LCL feeders). In general, the presence of feeder cells, especially LCL, allowed for a more consistent enrichment in $CD3^-CD56^+$ cells in A-LAK cultures from normal as well as patients' PBMNC.

**Figure 1** Growth of A-LAK cell cultures established from PBMNC of AML patients and normal individuals. A-LAK cells were cultured for 14 days without feeder cells, with ConA-stimulated PBL, or with LCL. The bars indicate medians. The asterisks indicate significant differences ($P < 0.002$) in comparison to A-LAK cells cultured without feeder cells.

**Figure 2** Percentages of cells expressing the NK-cell phenotype ($CD3^-CD56^+$) in A-LAK cell cultures established from PBMNC of patients or normal individuals and grown with or without feeder cells. The phenotypic markers were determined by two-colour flow cytometry in A-LAK cultures on day 14 of growth. The bars indicate medians. The asterisk indicates a significant difference ($P < 0.01$ for ConA-PBL; $P < 0.001$ for LCL) in comparison to A-LAK cells cultured without feeder cells.

In all A-LAK cultures, cells other than $CD3^-CD56^+$ were T cells ($CD3^+CD56^+$ or $CD3^+CD56^-$, see Table I). Monocytes were consistently below 2%. As has been shown before for IL2-activated NK cells, a high proportion of A-LAK cells was negative for the CD16 antigen (Nagler *et al.*, 1989). Also, A-LAK cells were $CD45RO^-$, $CD45RA^-$ and $CD29^+$ (data not shown).

*Cytotoxicity of A-LAK cell cultures in patients with AML*

A-LAK cell cultures generated from AML patients in the presence or absence of feeder cells generally displayed high levels of cytotoxicity against NK-sensitive cell line K562 as well as against NK-resistant Daudi targets (Figure 3), with no significant difference on a per cell basis between A-LAK cell cultures grown with or without feeder cells. There was also no statistically significant difference between the levels of antitumour cytotoxicity between normal and patient A-LAK cells grown in the presence of feeder cells (Figure 3).

Cytotoxicity against autologous leukaemic blasts was also determined in those cases, where autologous frozen bone marrow cells collected at the time of the diagnosis were available (Figure 4a). In one out of two cases, there was considerable cytotoxicity against autologous blasts (1,379 LU). Interestingly, these effectors failed to lyse three allogeneic AML-blasts (data not shown). In the second case, far less autologous cytotoxicity was found (11 LU). Cytotoxicity against cryopreserved allogeneic AML blasts were also measured in several other cases (Figure 4b). In general, cytotoxicity against allogeneic blasts was low, with a median of 40 LU and range of 19–1,768.

In 15 cases, A-LAK cells were tested for cytotoxicity against normal allogeneic bone marrow cells and found to be able to lyse these normal targets (median 504, range 10–2715

**Table I** Phenotypic characteristics of A-LAK cells cocultured with LCL or allogeneic mitogen-activated PBL as feeders[a]

| | | *% Positive cells* | | | | | |
|---|---|---|---|---|---|---|---|
| *Donors of A-LAK cells* | *Feeder cells* | *CD3⁻ CD56⁺* | *CD16⁻ CD56⁺* | *CD16⁺ CD56⁺* | *CD16⁺ CD56⁻* | *CD3⁺ CD56⁻* | *CD3⁺ CD56⁺* |
| AML patients ($n = 7$) | None | 51 ± 12 | 47 ± 13 | 16 ± 1 | 2 ± 1 | 35 ± 9 | 12 ± 3 |
| AML patients ($n = 7$) | ConA-PBL | 62 ± 9 | 48 ± 8 | 25 ± 1 | 2 ± 1 | 23 ± 9 | 8 ± 2 |
| AML patients ($n = 8$) | LCL | 77 ± 5 | 69 ± 5 | 13 ± 6 | 1 ± 0 | 12 ± 5 | 5 ± 10 |
| Normal ($n = 14$) | None | 53 ± 9 | ND | ND | ND | 18 ± 6 | 14 ± 4 |
| Normal ($n = 14$) | ConA-PBL | 80 ± 7[b] | 55 ± 15 | 43 ± 15 | 3 ± 2 | 7 ± 5 | 5 ± 2 |
| Normal ($n = 11$) | LCL | 90 ± 2[c] | 37 ± 11 | 60 ± 11 | 3 ± 2 | 3 ± 1 | 7 ± 2 |

[a]Two-colour flow cytometry was used to determine proportions of different lymphocyte subpopulations in A-LAK cells cocultured for 14 days with prestimulated and irradiated allogeneic PBL or LCL. Data are means ± s.e.m. [b]Significantly different from A-LAK cells of normal controls grown without feeder cells ($P<0.01$). [c]Significantly different from A-LAK cells of normal controls grown without feeder cells ($P<0.001$).

**Figure 3** Antitumour cytotoxicity of A-LAK cells in cultures established from patients with AML or normal volunteers. A-LAK cultures grown in the presence or absence of ConA-PBL or LCL as feeder cells for 14 days were tested in 4 h $^{51}Cr$-release assays against K562 and Daudi targets. The data are means ± s.e.m.

**Figure 4** Cytotoxicity of A-LAK cells obtained from AML patients against autologous fresh leukaemic blasts **a**, and allogeneic leukaemic blasts **b**, in comparison to cytotoxicity against K562 or Daudi targets. Cytotoxicity was tested in 4 h $^{51}Cr$-release assays.

LU/$10^7$ cells, see Figure 5). Nevertheless, the susceptibility of normal BM cells to lysis by A-LAK cells was 10 folds lower than that of Daudi (median 5240, range 2271–27661 LU/$10^7$ cells) and 22 folds lower than that of K562 (median 11350, range 5455–52885).

We also compared total lytic units (TLU) of cytotoxicity in cultures grown from patients' PBL with or without ConA-activated PBL or LCL as feeder cells (Figure 6). Total lytic activity against both K562 and Daudi in A-LAK cell cultures grown with feeder cells was significantly greater than that in paired cultures grown without feeders ($P<0.02$ for ConA-PBL; $P<0.005$ for LCL).

### *Quality of A-LAK cells generated from patients with AML*

Our results indicated that in spite of overall improvement in growth of A-LAK cells in the presence of feeders, some heterogeneity existed among patients in regard to the augmenting effects of feeder cells on expansion, phenotype and cytotoxicity of cultured effector cells. As a practical parameter to discriminate between patients with good and impaired ability to generate A-LAK cells *in vitro*, we arbitrarily defined 'good' A-LAK generation as: expansion ⩾ 100 fold with $CD3^-CD56^+$ cells ⩾ 50% and 'borderline' cultures as: expansion > 50 fold with $CD3^-CD56^+$ cells ⩾ 80%. Among eight A-LAK cultures grown from patients' PBMNC in the presence of ConA-stimulated feeders, four were considered 'good' and one 'borderline' (success rate of 62%; Table II). A similar success rate was obtained in presence of LCL feeders, where five 'good' out of eight A-LAK cultures were generated. None of the nine cultures grown without feeder cells met the above criteria. Thus, the addition of feeder cells resulted in a significant improvement of A-LAK generation in patients with AML ($P<0.02$ for both PBL and LCL feeders) from cryopreserved PBMNC. It may be important to note that 'good' *in vitro* responses occurred both among patients treated and not previously treated with chemotherapy (Table II).

**Figure 5** Cytotoxicity of A-LAK cells obtained from PBMNC of normal donors against normal bone marrow (BM) cells in comparison to cytotoxicity against K562 or Daudi targets. Cytotoxicity was measured in 4 h $^{51}Cr$ release assays. The bars indicate medians. The asterisk indicates a significant difference ($P < 0.0001$) between levels of cytotoxicity against tumour targets *vs* that against normal BM.

**Figure 6** Total lytic units of antitumour activity in A-LAK cultures from AML patients. A-LAK cells were cultured in the presence or absence of feeder cells for 14 days and then tested for cytotoxicity against K562 and Daudi targets. The bars are medians. The asterisks indicate significant differences ($P < 0.02$ for ConA-PBL; $P < 0.005$ for LCL) in comparison to A-LAK cells cultured without feeder cells.

**Table II** Quality of A-LAK cultures from patients with ANLL

| | | | *Generation of A-LAK cells*[a] | | |
|---|---|---|---|---|---|
| *Patients* | *Diagnosis* | *Stage of disease* | *No feeders* | *ConA-PBL feeders* | *LCL feeders* |
| P.T. | ANLL-M4 | Untreated | – | + | + |
| M.M. | ANLL-M4 | Untreated | nd | + | + |
| M.L. | ANLL-M3 | Untreated | – | + | – |
| K.F. | ANLL-M4 | Untreated | – | nd | – |
| E.C. | ANLL-M4 | Untreated | – | + | nd |
| D.P. | ANLL | Untreated | – | – | nd |
| T.K. | ANLL-M3 | Remission | – | nd | + |
| M.S. | ANLL-M3 | Remission | – | – | – |
| G.D. | ANLL-M2 | Remission | – | – | + |
| E.E. | ABKK-M2 | Remission | – | + | + |
| | AML patients | | 0/9 | 5/8[b] (62%) | 5/8[b] (62%) |
| | Normal controls | | 3/12 (25%) | 11/13 (84%) | 11/11[c] (100%) |

[a]'Good' (+) A-LAK cell cultures were defined as those with expansion >100 fold and CD3$^-$CD56$^+$ >50%; 'Borderline' (±) A-LAK cultures had expansion >50 fold and CD3$^-$CD56$^+$ >80%; 'negative' (–) cultures failed to generate A-LAK cells under conditions defined in Materials and methods. [b]The frequency .of 'good' or 'borderline' cultures was significantly increased compared to A-LAK witout feeder cells ($P < 0.01$). [c]The frequency of 'good' or 'borderline' cultures was significantly greater than in AML patients ($P < 0.005$).

### Discussion

NK cells appear to play an important role in defence against cancer, including leukaemia. It has been observed that NK activity in patients with preleukaemic disorders or with leukaemia in acute stage or in relapse is often decreased (Lotzova *et al.*, 1986; Pizzolo *et al.*, 1988; Whiteside & Herberman, 1989). In contrast, NK activity tends to return to normal in remission (Lotzova *et al.*, 1986; Pizzolo *et al.*, 1988). These observations suggested that there exists a correlation between NK activity and the disease activity in acute leukaemia. Also, NK cells from patients with leukaemia (acute and chronic, lymphocytic and myelogenous) have been shown to be impaired in their ability to bind to and lyse tumour cells, in their recycling capacity, and in production of NK cytotoxic factor (Lotzova, 1984; Lotzova *et al.*, 1986). These defects can be, at least in part, reversed *in vitro* by the addition of IL-2 (Lotzova *et al.*, 1987). On the basis of such *in vitro* observations, it has been suggested that AIT with IL-2 activated NK cells may be effective in control of lymphoproliferative disorders. A-LAK cells could be used for AIT of human leukaemias, if available in numbers sufficient for therapy.

Our data demonstrate the feasibility of generating large quantities of highly cytotoxic A-LAK cells from cryopreserved peripheral blood mononuclear cells (PBMNC) or patients with untreated as well as remitted acute myelogenous leukaemia (AML). In this disease, A-LAK generation in the presence of IL-2 and conditioned medium only, as originally described by Melder *et al.* (Melder *et al.*, 1988), is generally poor. In order to improve and optimise this technique for cells of patients with AML, ConA-prestimulated allogeneic PBL or EBV-transformed allogeneic LCL were added as feeder cells to patient PBMNC cultures in the presence of IL-2. This approach has been shown by us recently to improve A-LAK generation in both normal individuals (Rabinowich *et al.*, 1991) and patients with solid cancers (Sedlmayr *et al.*, 1991). The addition of feeder cells to cryopreserved patient PBMNC increased *in vitro* proliferation of these cells to levels which were significantly greater than those in paired cultures grown without feeders and as good as those of normal PBMNC grown under comparable conditions. In addition to increasing proliferation, the presence of feeder cells also seemed to improve consistency of obtaining cultures highly enriched in CD3$^-$CD56$^+$ cells. It is unclear by what mechanism feeder cells augment A-LAK cell proliferation. We hypothesise that they provide growth factors necessary for successful expansion of enriched NK cells *in vitro*.

A number of investigations have demonstrated that LAK activity can be generated from PBMNC of leukaemic patients in remission (Adler *et al.*, 1988; Adler *et al.*, 1989; Findley *et al.*, 1988; Tahara *et al.*, 1988; Teichmann *et al.*, 1989), and, on the basis of these preclinical studies, immunotherapy with rIL2 is being used in patients with acute leukaemia (Foa *et al.*, 1990*a*; Foa *et al.*, 1990*c*; Gottlieb *et al.*, 1989). The main intent of such immunotherapy is to eliminate minimal residual disease through *in vivo* activation of LAK cell precursors (Gottlieb *et al.*, 1989). More recent studies seem to indicate, however, that autologous LAK cells might not be as effective for this purpose as originally expected, because LAK activity, as well as NK activitiy, is impaired in leukaemia patients at presentation (Foa *et al.*, 1990*b*). It appears that both defective lytic activity of the effectors and resistance of leukaemic blasts to lysis by these effectors may be responsible for this impairment (Foa *et al.*, 1991). Indeed, our study illustrates that patients with acute nonlymphocytic leukaemia (ANLL) at presentation or in remission are not able to generate IL-2 activated NK effector cells unless special *in vitro* conditions are provided. This might indicate that the impairment in effector cell generation and/or function is reversible when NK cells are separated from putative suppressor cells or factors and provided with necessary growth factors *in vitro*.

Successful generation of A-LAK cells from patients in the chronic phase of chronic myelogenous leukaemia (CML) has been recently reported (Verfaillie *et al.*, 1989). As compared to unseparated LAK cells from the same patients, these A-LAK cells demonstrated superior antitumour cytotoxicity *in vitro*. Importantly, these A-LAK cells were Philadelphia chromosome-negative and did not show the *bcr* gene rearrangement in contrast to the leukaemic cells in the same patients. In a more recent study, Verfaillie *et al.* (Verfaillie *et al.*, 1990) reported that proliferation of A-LAK cells from patients with CML was significantly increased compared to A-LAK cells from normal volunteers, but the ability to proliferate *in vitro* declined in parallel with the progression of the disease. When added to our observations, these data strongly suggest that it is feasible to generate sufficient numbers of A-LAK cells for AIT in patients with leukaemias especially in early disease and in remission.

Considerable information has been acquired regarding lysis of leukaemic blasts by LAK cells. Most leukaemic cell lines have been shown to be susceptible *in vitro* to lysis by LAK cells. However, killing of these cell lines does not appear to correlate with cytotoxicity against autologous leukaemic blasts (Foa *et al.*, 1991). While fresh leukaemic blasts are generally sensitive to LAK cell lysis, most are resistant to unstimulated NK cells (Adler *et al.*, 1988; Adler *et al.*, 1989; Fierro *et al.*, 1988; Findley *et al.*, 1988; Lotzova *et al.*, 1987; Oshimi *et al.*, 1986; Panayotides *et al.*, 1988; Tahara *et al.*, 1988; Foa *et al.*, 1991). Also, inherent resistance of leukaemic blasts to LAK-mediated lysis has been observed in acute leukaemic patients with active disease (Foa *et al.*, 1991). To a limited extent, it was possible in this study to check effectiveness of patients' A-LAK cells against fresh cryopreserved allogeneic AML blasts. These leukaemic targets were variably sensitive to lysis by A-LAK cells, with some fresh targets showing considerable sensitivity. It has been suggested that the presence of LAK activity against autologous blasts may be a useful marker of disease (Foa *et al.*, 1991). In two cases, we tested fresh AML blasts for lysis by autologous A-LAK cells, and demonstrated good levels of activity in one case. Overall, autologous killing by A-LAK cells may be a more useful measure of antitumour immunity than lysis of leukaemic cell lines. Although the addition of feeder cells to A-LAK cells did not significantly augment their cytotoxicity against K562, and Daudi (on a per cell basis), due to increased proliferation of A-LAK cells in the presence of feeder cells, TLU of anti-K562 and anti-Daudi activity were significantly increased. Further experiments need to be performed to investigate the possibility that feeder cells might alos improve *in vitro* generation of A-LAK cells with high levels of cytotoxicity against allogeneic and autologous leukaemic blasts.

LAK cells have been shown to have little cytotoxicity against normal bone marrow cells (Van den Brink *et al.*, 1989; Van den Brink *et al.*, 1990). In competitive inhibition assays, normal bone marrow cells failed to compete with LAK effectors for killing of hematopoietic tumour cell lines, K562 and HL60, or fresh-frozen AML blasts (Trinchieri *et al.*, 1987). Furthermore, LAK cells had no inhibitory effect on hematopoietic growth in colony forming assays (Van den Brink *et al.*, 1989). Nevertheless, a concern that IL-2 activated effectors may damage normal bone marrow cells exists, because of evidence that NK cells might suppress hematopoietic stem cell function *in vitro* (Trinchieri *et al.*, 1987). In particular, inhibition of clonogenic growth of human AML stem cells by LAK cells has been reported (Lista *et al.*, 1989). As A-LAK cells derived from PBMNC of normal volunteers have significantly higher antitumour cytolytic activity on a per cell basis than LAK cells, the possibility exists that A-LAK cells may cause adverse effects on normal hematopoietic progenitor cells. Our observation that, in 15 cases, normal bone marrow cells were lysed by A-LAK cells appears to be important and deserves further studies. However, the highly significant difference observed in sensitivity to the cytolytic activity of A-LAK cells between tumour cell lines (K562 or Daudi) and normal bone marrow cells suggest that A-LAK preferentially kill tumour cells. This difference in susceptibility to lysis by A-LAK cells can perhaps be used to advantage in designing a therapeutic protocol that would optimise the ability of A-LAK cells to kill leukaemic cells and yet diminish damage to normal bone marrow elements. If, however, A-LAK cells prove to be highly toxic to hematopoietic progenitor cells, the rationale for AIT with A-LAK cells in patients with leukaemia may have to be reevaluated.

In summary, we have demonstrated that generation of highly homogeneous populations of IL2-activated NK cells from cryopreserved PBMNC is feasible in patients with AML, especially since use of irradiated, allogeneic, ConA-activated feeder cells significantly enhances proliferation of A-LAK cells, increases consistency of obtaining cultures highly enriched in $CD3^-CD56^+$ effectors and augments total anti-tumour activity.

P. Sedlmayr is a recipient of a fellowship grant from the Max Kade - Foundation.

**References**

ADLER, A., CHERVENICK, P.A., WHITESIDE, T.L., LOTZOVA, E. & HERBERMAN, R.B. (1988). Interleukin 2 induction of lymphokine-activated killer (LAK) activity in the peripheral blood and bone marrow of acute leukemia patients. I. Feasibility of LAK generation in adult patients with active disease and in remission. *Blood*, **71**, 709.

ADLER, A., ALBO, V., BLATT, J., WHITESIDE, T.L. & HERBERMAN, R.B. (1989). Interleukin 2 induction of lymphokine-activated killer (LAK) activity in the peripheral blood and bone marrow of acute leukemia patients II. Feasibility of LAK generation in children with active disease and in remission. *Blood*, **74**, 1690.

FIERRO, M.T., LIAO, X.S., LUSSO, P. & 6 others. (1988) *In vitro* and *in vivo* susceptibility of human leukemic cells to lymphokine activated killer activity. *Leukemia*, **2**, 50.

FINDLEY, H.W.J., MAGEED, A.A.JR., NASR, S.A. & RAGAB, A.H. (1988). Recombinant interleukin-2 activates peripheral blood lymphocytes from children with acute leukemia to kill autologous leukemic cells. *Cancer*, **62**, 1928.

FOA, R., MELONI, G., TOSTI, S. & 7 others (1990*a*). Treatment of residual disease in acute leukemia patients with recombinant interleukin 2 (IL-2): clinical and biological findings. *Bone Marrow Trans.*, **6**, 98.

FOA, R., FIERRO, M.T., RASPADORI, D. & 9 others (1990*b*). Lymphokine activated killer (LAK) cell activity in B and T chronic lymphoid leukemia: defective LAK generation and reduced susceptibility of the leukemic cells to allogeneic and autologous LAK effectors. *Blood*, **76**, 1349.

FOA, R., FIERRO, M.T., TOSTI, S., MELONI, G., GAVOSTO, F. & MANDELLI, F. (1990*c*). Induction and persistence of complete remission in a resistant acute myeloid leukemia patient after treatment with recombinant interleukin 1. *Leukemia & Lymphoma*, **1**, 113.

FOA, R., FIERRO, M.T., CESANO, A., GUARWI, A., BENFERRONI, M., RASPADORI, D., MINIERO, R., LAURIA, F. & GAVOSTO, F. (1991). Defective lymphokine-activated killer cell generation and activity in acute leukemia patients with active disease. *Blood*, **78**, 1041.

GOTTLIEB, D.J., BRENNER, M.K., HESLOP, H.E. & 8 others. (1989). A phase I clinical trial of recombinant interleukin 2 following high-dose chemoradiotherapy for maematological malignancy: applicability to the elimination of minimal residual disease. *Br. J. Cancer*, **60**, 610.

GRIMM, E.A., MAZUMDER, A., ZHANG, H.Z. & ROSENBERG, S.A. (1982). Lymphokine activated killer phenomenon: I. Lysis of natural killer resistant fresh solid tumor cells by interleukin-2 activated autologous human peripheral blood lymphocytes. *J. Exp. Med.*, **155**, 1823.

HERBERMAN, R.B., HISERODT, J.C., VUJANOVIC, N. & 11 others (1987). Lymphokine-activated killer cell activity. Characteristics of effector cells and their progenitors in blood and spleen. *Immunol. Today*, **8**, 178.

ITOH, K., TILDEN, A.B. & BALCH, C.M. (1986). Lysis of human solid tumor cells by lymphokine-activated natural killer cells. *J. Immunol.*, **136**, 3910.

LAFRENIERE, R. & ROSENBERG, S.A. (1985). Successful immunotherapy of murine experimental hepatic metastases with lymphokine-activated killer cells and recombinant interleukin 2. *Cancer Res.*, **45**, 3735.

LISTA, P., FIERRO, M.T., LIAO, X.S. & 5 others (1989). Lymphokine-activated killer (LAK) cells inhibit the clonogenic growth of human leukemic stem cells. *Eur. J. Haematol.*, **42**, 425.

LOTZOVA, E. (1984). The role of natural killer cells in immune surveillance against malignancies. *Cancer Bull.*, **36**, 215.

LOTZOVA, E., SAVARY, C.A. & HERBERMAN, R.B. (1986). Impaired NK cell profile in leukemia patients. In *Immunology of Natural Killer Cells. Vol. II.*, Lotzova, E. & Herberman, R.B. (eds) p. 29, Karger: Basal.

LOTZOVA, E., SAVARY, C.A. & HERBERMAN, R.B. (1987). Induction of NK cell activity against fresh human leukemia in culture with interleukin 2. *J. Immunol.*, **138**, 2718.

MELDER, R.J., WHITESIDE, T.L., VUJANOVIC, N.L.,HISERODT, J.C. & HERBERMAN, R.B. (1988). A new approach to generating antitumour effectors for adoptive immunotherapy using human adherent lymphokine-activated killer-cells. *Cancer Res.*, **48**, 3461.

MULE, J.J., SHU, S., SCHWARZ, S.L. & ROSENBERG, S.A. (1984). Adoptive immunotherapy of established pulmonary metastases with LAK cells and recombinant interleukin-2. *Science (Wash. DC)*, **225**, 1487.

NAGLER, A., LANIER, L.L., CWIRLA, S. & PHILLIPS, J.H. (1989). Comparative studies of human FcRIII-positive and negative natural killer cells. *J. Immmunol.*, **143**, 3183.

ORTALDO, J.R., MASON, A. & OVERTON, R. (1986). Lymphokine-activated killer cells: analysis of progenitors and effectors. *J. Exp. Med.*, **164**, 1193.

OSHIMI, K., OSHIMI, Y., AKUTSU, M. & 4 others (1986). Cytotoxicity of interleukin 2-activated lymphocytes for leukemia and lymphoma cells. *Blood*, **68**, 938.

PANAYOTIDES, P., PORWIT, A., SJOGREN, A.M., WASSERMAN, J. & REIZENSTEIN, P. (1988). Resistance of some leukemic blasts to lysis by lymphokine activated killer (LAK) cells. *Eur. J. Hematol.*, **40**, 362.

PHILLIPS, J.H. & LANIER, L.L. (1986). Dissection of the lyphokine-activated killer phenomenon: relative contribution of peripheral blood natural killer cells and T lymphocytes to cytolysis. *J. Exp. Med.*, **164**, 814.

PIZZOLO, G., TRENTIN, L., VINANTE, F. & 12 others (1988). Natural killer cell function and lymphoid subpopulations in acute non-lymphoblastic leukemia in complete remission. *Br. J. Cancer*, **58**, 368.

PROSS, H.F., BAINES, M.T., RUBIN, P., SHRAGG, E.P. & PATTESON, M.S. (1981). Spontaneous human lymphocyte mediated cytotoxicity against tumor target cells. IX. The quantitation of natural killer cell activity. *J. Clin. Immunol.*, **1**, 51.

RABINOWICH, H., SEDLMAYR, P., HERBERMAN, R.B. & WHITESIDE, T.L. (1991). Increased proliferation, lytic activity and purity of natural killer cells co-cultured with mitogen-activated feeder cells. *Cellular Immunol.*, **135**, 454.

ROSENBERG, S.A., LOTZE, M.T., MUUL, L.M. & 7 others (1985). Observations on the systemic administration of autologous lymphokine-activated killer cells and recombinant interleukin-2 to patients with metastatic cancer. *N. Engl. J. Med.*, **314**, 1485.

ROSENBERG, S.A., LOTZE, M.T., MUUL, L.M. & 10 others (1987). A progress report on the treatment of 157 patients with advanced cancer using lymphokine-activated killer cells and interleukin-2 or high-dose interleukin-2 alone. *N. Engl. J. Med.*, **316**, 889.

SACCHI, M., VITOLO, D., SEDLMAYR, P. & 4 others (1991). Induction of tumor regression in experimental model of human head and neck cancer by human A-LAK cells and IL2. *Int. J. Cancer*, **47**, 784.

SCHWARZ, R.E., VUJANOVIC, N.L. & HISERODT, J.C. (1989). Enhanced antimetastatic activity of lymphokine-activated killer cells purified and expanded by their adherence to plastic. *Cancer Res.*, **49**, 1441.

SEDLMAYR, P., RABINOWICH, H., ELDER, E.M. & 4 others (1991). Depressed ability of patients with melanoma of renal cell carcinoma to generate adherent lymphokine activated killer (A-LAK) cells. *J. Immunother.*, (in press).

TAHARA, T., ISEKI, R., MORISHIMA, Y., YOKOMAKU, S., OHNO, R. & SAITO, H. (1988) Generation and characterization of lymphokine-activated killer cells against fresh human leukemia cells. *Jpn. J. Cancer Res.*, **79**, 390.

TEICHMANN, J.V., LUDWIG, W.D., SEIBT-JUNG, H. & THIEL, E. (1989). Induction of lymphokine-activated killer cells against human leukemia cells *in vitro*. *Blood*, **59**, 21.

TRINCHIERI, G., MURPHY, M. & PERUSSIA, B. (1987). Regulation of hematopoiesis by T lymphocytes and natural killer cells. In *CRC Critical Reviews in Oncology and Hematology*. Davis, S. (ed.) **7**, 219. CRC Press: Boca Raton, FL.

VAN DEN BRINK, M.R.M., VOOGT, P.J. MARIJT, W.A.G., VAN LUXEMBURG-HEYS, S.A.P., VAN ROOD, J.J. & BRAND, A. (1989). Lymphokine-activated killer cells selectively kill tumor cells in bone marrow without compromising bone marrow stem cell function *in vitro*. *Blood*, **74**, 354.

VAN DEN BRINK, M.R.M., VOOGT, P.J., LONG, G.S., CRAMER, D.V. & HISERODT, J.C. (1990). LAK cells and autologous bone marrow transplantation: towards a cure for leukemia. In *Interleukin 2-Activated Killer Cells*, Lotzova, E. & Herberman, R.B. (eds) p. 219. CRC Press: Boca Raton, FL.

VERFAILLIE, C., MILLER, W., KAY, N. & MCGLAVE, P. (1989). Adherent lymphokine-activated killer cells in chronic myelogenous leukemia; a benign cell population with potent cytotoxic activity. *Blood*, **74**, 793.

VERFAILLE, C., KAY, N., MILLER, W. & MCGLAVE, P. (1990). Diminished A-LAK cytotoxicity and proliferation accompany disease progression in chronic myelogenous leukemia. *Blood*, **76**, 401.

WHITESIDE, T.L., ERNSTOFF, M.S., NAIR, S., KIRKWOOD, J.M. & HERBERMAN, R.B. (1990). *In vitro* generation and *in vivo* effects of adherent-lymphokine activated killer (A-LAK) cells and IL2 in patients with solid tumors. In *Natural Killer Cells: Biology and Clinical Application*, Schmidt, R.E. (ed.) p. 293. 6th Int. NK Cell Workshop, Goslar; Karger: Switzerland.

WHITESIDE, T.L., BRYANT, J., DAY, R. & HERBERMAN, R.B. (1990). Natural killer cytotoxicity in the diagnosis of immune dysfunction: criteria for a reproducible assay. *J. Clin. Lab. Analysis*, **4**, 102.

WHITESIDE, T.L. & HERBERMAN, R.B. (1989). The role of natural killer cells in human disease. *Clin. Immunol. Immunopath.*, **53**, 1.